# Romanian young adult perceptions on using heated tobacco products following exposure to direct marketing methods

**DOI:** 10.1038/s41533-023-00333-y

**Published:** 2023-03-02

**Authors:** Sergiu Chirila, Adriana Antohe, Cristina Isar, Catalina Panaitescu, Alice Malpass

**Affiliations:** 1 Faculty of Medicine, Ovidius University, Constanta, Romania; 2 Romanian Primary Care Respiratory Group RespiRO, Bucharest, Romania; 3 NIHR Applied Research Collaboration West (NIHR ARC West), Bristol, UK

**Keywords:** Lifestyle modification, Health policy, Public health

## Abstract

Heated tobacco products have a rapid uptake, especially among young people, mostly where advertising is unregulated, as is the case in Romania. This qualitative study explores the influence of direct marketing methods of heated tobacco products on young people, their perception and behaviour towards smoking. We have carried out 19 interviews with smokers of heated tobacco products (HTPs) or/and combustible cigarettes (CCs) or non-smokers (NS), aged 18–26. Using the thematic analysis, we have identified three overarching themes: (1) people, places, and subjects of marketing, (2) engagement with risk narratives and (3) social body, family bonds, and autonomous self. Even if most of the participants have been exposed to a mix of marketing methods, they did not acknowledge the influence that marketing has on their decision to experience smoking. Young adults’ decision to use heated tobacco products seems to be influenced by a cluster of reasons: overcoming the legislation gap which prohibits indoor use of combustible cigarettes but not heated tobacco products; the attractivity of the product (novelty, inviting appearance, technological appeal and price) and presumed less damaging effects on health.

## Introduction

In the last decade, heated tobacco products (HTPs) have been introduced and marketed, globally^1^, as offering a safer alternative to combustible cigarettes (CCs). Even though, at the European level, advertising for CCs is strictly regulated^2^, when referring to HTPs, there is a high heterogeneity in the way EU Directives are implemented in each country, resulting in different levels of control^3^. In Romania, the usage of tobacco products and their marketing are regulated through several distinct laws^4–6^. In Romania, smokeless tobacco products are defined as products that deliver nicotine without involving a combustion process. The Romanian legislation stipulates that indoor smoking is prohibited only if the tobacco is burned, leaving people free to use HTPs indoors^4^. In Romania, there are two brands of HTPs currently available, IQOS and glo^7^, which were introduced on the local market in 2015 and 2017, respectively.

In a study, which compared data from six European countries, the authors identified an important increase in the awareness and use of HTPs among European cigarette smokers, in less than two years^8^, Romania being one of the countries with the highest ever use and current use of HTPs in 2018, with a 4% ever use of HTPs. Also, in another study^9^, with data collected in 2017 and 2018, that compared the prevalence of HTPs usage among 11 European countries, Romania was second after Greece, with 3% (CI 95% 2.2-3.8) of participants being ever users of HTPs and 1.3% (CI 95% 0.9–1.7) declaring to be current users. When data were analysed according to age groups, the highest prevalence was among young adults aged 15 to 25. More recent data, from 2020, indicate that for Romania, the prevalence of ever use of HTPs increased to 5.2 (95% CI 4.2–6.9), while the current use was estimated to be 0.5% (95% CI 0.2–1.2)^10^.

Growing research is being conducted on discovering the factors that influence the initiation and use of HTPs as well as product knowledge^9,11–14^, yet no previous qualitative study on HTPs has specifically examined young adults. In a qualitative study by Tompkins, conducted in the United Kingdom, there were six main factors (health, financial, physical, practical, psychological, and social) that influenced the initiation and use of HTPs^15^. Another qualitative study, conducted in Japan and Switzerland, concluded that cultural factors appear to affect the appeal of the marketing methods used, indicating that uptake will likely be different from country to country^16^.

At the same time, studies that analyse marketing methods focus on the strategies used by the producers to increase the number of HTPs consumers. It seems that HTP producers use every marketing channel available for advertising: from speciality stores^17,18^, easily accessible in all major stores in countries where HTPs are marketed, to internet platforms^19^, where the role of social “influencers” and the idea of community^20,21^ are widely used as marketing strategies. Advertising and sales of HTPs via the internet are accessible even in countries where these products are illegal^19,22^.

The rapid uptake of HTPs (particularly amongst the younger generations), which is likely to be linked to the unregulated marketing strategies and the high availability of the products, raises serious public health concerns.

The purpose of our qualitative study was to identify the effects of direct marketing of HTPs on young adults in Romania, including ascertaining the spaces and places where this marketing activity is encountered. The research team was also keen to know what impact, if any, social contexts (family, friends, and work colleagues) had on decisions to use HTPs. Finally, the research team was interested in establishing the relationship between HTPs and combustible smoking habits and decision making, for example - if using HTPs was the first step in taking up combustible cigarettes, or not, and if HTPs replaced or were used alongside combustible cigarettes.

## Methods

### Study design

The interviews reported in this paper are part of a mixed-methods study. We started with a survey to initially explore the topic with a larger sample of young adults and to help inform recruitment sampling and the development of the topic guide.

Ethics approval for conducting the study was obtained from the Bioethics Committee of University “Ovidius” of Constanta (No. 19607/31.12.2019).

### Recruitment and consent for interviews

To identify potential candidates, we designed an online questionnaire with 21 free text questions which was distributed by advertising a hyperlink via online student groups, social networks, as well as displaying it on local posters on a University campus in Constanta county likely to be seen by young adults aged between 18 and 26. The questionnaire allowed us to identify potential candidates for the interviews, based on their age, smoking status, use of CCs, HTPs and willingness to participate in the interview. Most of the answers were gathered in February 2020, just before the epidemiological context changed due to the SARS-CoV-2 pandemic. Based on purposive sampling, participants that gave their consent were invited to take part in an interview. Due to the pandemic, a lag occurred between the survey and the time of the interviews, resulting in some survey participants not being available to participate in the interviews. To complete the study sample, we decided to recruit more participants through direct contact, without prior participation in the questionnaire. These participants were invited to the interviews based on snowball sampling.

### Sampling strategy and sample size

We aimed to recruit between 25 and 30 young adults for the interview study, using a purposive sampling strategy, and we obtained the consent to participate in the study from the necessary number of possible subjects. Participants need to be aged 18–26, living in Romania and fall into one of three types of users: CC smokers, HTP users or non-smokers.

Participants that declared to have used HTPs or smoked CC during the last 30 days, with a frequency higher than once per week were considered HTP users or CC smokers. Young adults that declared that they only tried on occasions CC or HTPs were considered as being non-smokers.

Some of the HTPs users were also smoking CCs, therefore we decided to label this category as MIX and to analyse it separately. This aspect was important as the perspective of dual use had the potential to offer new insights, different from the ones of exclusive HTPs or CC smokers.

This stratification of the participants based on their tobacco use status and type was implemented to obtain a large and detailed palette of opinions as possible.

### Data collection

Due to the epidemiological conditions, all interviews were conducted online between May 2020 to February 2021 by either A.A., C.I. or C.P. All participants provided written informed consent for their interviews to be recorded and for the data to be used in this study.

A topic guide was developed based on the survey responses and a literature review and then piloted on three interviewees. The survey allowed us to gain insight on aspects related to tobacco consumption among young adults and their interaction with tobacco promotional teams or other forms of tobacco marketing, aspects that were investigated during the following interviews.

The topic guide covered the smoking history of the participants (including the context of first smoking experiences), the perceptions and experiences related to HTPs marketing and use, as well as the perceived relationship between HTPs use and health. The topic guide was flexible enough to allow exploration of issues raised by the participant. We stopped at 19 interviews, from the planned 25–30, because we felt that data saturation with respect to our aim was achieved.

Interviews lasted between 17 and 70 min (median 39 minutes). Interviews with non-smokers tending towards of being shorter when compared to the interviews conducted on participants from other tobacco use status (a median duration of 23 min for non-smokers and 39.5 min for participants from other tobacco use categories).

All the interviews were recorded and transcribed verbatim by the research team.

### Data analysis

The interviews were held in Romanian - the native language of the bi-lingual researchers. The first four interviews were transcribed verbatim from the audio file initially into Romanian and then into English. This was to allow the only English-speaking member of the research team who was advising on qualitative methods to be involved in the initial stages of coding. The transcripts for the first four interviews were imported into NVivo 12 and were coded independently by all members of the research team. At this stage, the Romanian team members worked on the English transcript, but referred back continuously to the Romanian transcript to check the meaning was not lost in translation.

The initial coding framework was formulated in English and then used for the subsequent 15 interviews. The 15 transcripts were then coded by the Romanian members of the team, without prior translation into English. Decisions to work in this way were informed by the literature on translation during qualitative research^23,24^. Each interview was coded, independently, by two researchers, and lists of codes for each transcript were analysed and any differences were resolved by discussions within team meetings. The development of codes into overarching themes and sub-themes was discussed with the whole team. Analysis of the codes was supported by mapping patterns in how codes were attributed to different participants as well as mapping the relationships between codes. A thematic approach was used for analysis, including the interpretative arrangement of codes into thematic patterns, which were discussed and finalised by the research team. This is often referred to as emergent thematic analysis^25^.

### Reporting summary

Further information on research design is available in the Nature Research Reporting Summary linked to this article.

## Results

The study population consisted of young people, 18–26 years old, from Romania, exclusive CC users (*n* = 5), exclusive HTP users (*n* = 4), dual CC + HTP users (MIX; *n* = 5), and non-smokers (*n* = 5). See Table 1 for participant characteristics.

**Table 1:** Participant’s characteristics.

Demographic	Exclusive CC (*n* = 5)	MIX (CC + HTP) (*n* = 4)	Exclusive HTP (*n* = 5)	NS (*n* = 5)
Age (years)				
18–22	0	2	3	4
23–26	5	2	2	1
Mean age ± standard deviation	23.6 ± 2.79	22.75 ± 2.21	21.8 ± 2.77	20.8 ± 2.16
Median age	25	23	21	21
Sex				
Female	5	3	5	3
Male	0	1	0	2
Occupation				
Highschool student	0	0	0	1
University student	4	3	3	4
Employed	1	1	2	0
Education domain				
Medicine	2	3	3	4
Other	3	1	2	1

We present the results from our thematic analysis in relation to three over-arching themes and 12 sub-themes that impact the decision to start using HTPs. 1. People, places and subjects of marketing; 2. Engagement with risk narratives; and 3. Social body, family bonds and autonomous self. Table 2 shows the complete list of themes and sub-themes.

**Table 2:** Over-arching themes and sub-themes.

Overarching themes	Sub-themes
People, places, and subjects of marketing HTPs	Product as badge
	Price – the financial hook
	Place – the universe
	Promotion – the start of a dialogue
	People as influencers
Engagement with risk narratives	Smoking is about losing health
	Harmful effects of smoking - doubted, dismissed, minimised, or avoided
	Perceptions of addiction
	Risk narratives surrounding HTPs
Social body, family bonds, and autonomous self	Individuality - autonomous self
	Family bonds
	Social Body

Themes are outlined below with the use of verbatim quotes. Data extracts are tagged with a unique anonymised identifier indicating the sex of the participant (F for female, M for male), the age and the current smoking status (CC – combustible cigarettes, HTP – heated tobacco products users, MIX – users of both combustible cigarettes and heated tobacco products, NS – non-smoker), for example, F26-CC. Because in the case of two participants, the code was identical, we added 1 or 2 at the end of the tag.

### People, places and subjects of marketing

In our interviews, young adults experienced elements of almost all the P’s of the tobacco marketing mix: Product, Price, Place, Promotion, People, Process and Physical evidence^26^.

They consider themselves above marketing influence (the decision to start smoking, they say, is theirs), yet they manifest interest in new devices and news.

“I didn’t pay attention to marketing and advertising, and I don’t think that my peer opinion was important, either. I mean it was something about me, to see what it was about. No, I wasn’t influenced, maybe on an unconscious level this could be possible.” (F25-MIX)

Marketing is nowadays greatly impacted by the social sciences, especially psychology^27^. There are also de-marketing strategies that discourage smoking^28^, from all of the smoking continuum (stopping smoking and preventing starting smoking).

In our data, we explore five separate elements of tobacco marketing.

### Product as badge

Tobacco has been called the ultimate “badge product”^29^ for it sends an implicit message, repeated every time young adults join social reunions:

“this is another reason I continued to smoke: I felt it became my thing, my signature.” (F25-CC1)

Moreover, being an early adopter is another way of being distinctive but alike to the’chosen’ ones: the daring and courageous. The HTP devices have nice designs, and many functions that stimulate interaction (i.e. connection with the smartphone). Some are singled out as elegant, this particular quality was believed to “set them apart” (F19-HTP):

“It appeared… 3 years ago, I was among the first ones to buy and use it…it was a pleasant experience from the start. It continued to be pleasant, I am a loyal client. In time there have been different devices …they did not attract me at all, IQOS has a more elegant design” (F26-HTP)

The package design and brand are visible and project a message of power and engagement, which can either turn young adults on or off certain products:

“I don’t like the design of glo, it’s big and ugly” (F19-HTP)

Size, graphics and colour are not a new marketing strategy for transferring symbolised attributes to HTP; it was there to enlist smokers of CC, right from the beginning:

“I remember buying my first pack of cigarettes, they were coloured, each cigarette had its own colour and I think that is what got me hooked.” (F19-HTP)

### Price – the financial hook

This is not direct marketing but it is a great hook to start discussing with people to get their attention.

When young adults start experimenting, price is not very important, for they need the image conveyed by the “badge”, but, in time, they are interested in lowering the price by choosing discounts, as “the cost is huge” (*F24-MIX):*

“Promotions for cartons, there are discounts, devices that are more colourful, look more… more beautiful” (F26-HTP)

Although young adults in our study knew about HTP-related contests and raffles, some “have never participated” (F26-HTP).

Young adults’ main financial hooks were gratuity packages, or buying the HTP device in installments “you can buy it for like 20 RON/month” (F19-HTP):

“the most attractive method is to receive a free package without buying anything… sometimes it’s also interesting to buy a package and get another one for free” (M20-MIX)

Not every participant in our study was susceptible to HTP marketing tactics, some perceived them as inappropriate, encouraging the increased use:

“I felt weird, for they made you buy a package for getting one for free, and I said….”I already have cigarettes”…. I don’t have to stockpile with cartridges right away, it seems inappropriate” (F25-CC2)

Young adults knew they were being manipulated by the idea of getting something for free when, actually, they were just buying in bulk (HTP companies overcome buyer uncertainty by giving HTP devices out for free when young adults buy a certain number of packages).

“you get a lot of free packs…they’re not really free but it’s the idea.” (F19-HTP).

Some use both CCs and HTPs and needed the advantage of the HTP’s price:

“[HTPs] are more accessible. The price of cigarettes rose… but the prices for these [HTPs] cigarette packages for these devices remained the same, didn’t increase…and for some students this difference counts” (M20-MIX)

### Place – the universe

Young adults in our study noticed HTPs advertising in gas stations, kiosks, hypermarkets, neighbourhood stores and coffee houses. This advertising presence aimed to normalise HTP use. It is also in public spaces that sales teams approach potential customers. Young adults described the approach as direct:

“in small shops, there are always tobacco representatives… ‘Do you smoke?’ …and if you say ‘Yes’, they make you different offers” (F19-HTP)

Young adults also compared online versus face-to-face marketing.

“I access the [HTP supplier] internet site… when I need to buy… I order and I get it delivered… I am unsubscribed from receiving emails, I only receive ads” (F26-HTP)

Participants noticed the unequal distribution of supplies:

“in the city (…) I find these products in 95% of the stores, but if I want to go on holiday…in a rural area, we cannot find these packages for respective devices” (M20-MIX)

### Promotion – the start of a dialogue

Young adults engage in conversation with tobacco sales teams:

“if you stay and get to answer a questionnaire, they give you small tokens: a lighter, a package of cigarettes or discount to cigarettes…” (F19-HTP)

Young adults observed the subtle ways HTP promoters succeeded in making an impression by sampling tobacco products in restaurants, recreational venues and events (experiential marketing) or through the internet and cartoons:

“I remember that from 3 years ago when I was a minor and I went to a festival… as a minor you are bombarded with ads”. (F19-HTP)

“the problem with this promotion is that it is a very, very subtle one, but has an effect. We don’t see cigarette commercials on TV, because it’s a matter of ethics…but still, we have the internet, lots of ways left…including cartoons” (M21-NS)

Some non-smoker young adults are aware that HTP is presented as less harmful for health, but see this as a marketing strategy.

“From what I have seen, many people have the impression that IQOS is less harmful” (M21-NS)

Improved sensory experience relating to smell and taste represents one of the main promotional messages, thus becoming important for young adults who perceive HTPs as lacking any “ugly smell” (F26-HTP):

“those [HTPs] have no smell, you could use them anywhere, in house, car or any enclosed space and there is no residual smell in the clothes” (F24-MIX)

HTP frees young adults from the problem of smoking in enclosed spaces (that is banned) and allows them the flexibility not to be ‘found out’ if they wanted to conceal a smoking habit from their family.

“it was quite hard and uncomfortable [smoking indoors being banned]… with IQOS we got rid of this problem, since I use [HTP] I’ve almost forgotten this problem that normal cigarettes smokers have” (F26-HTP)

“you can smoke it anywhere and I am hiding from the family…if somebody catches me I close it quickly and put it in my pocket, it’s more flexible somehow” (F24-MIX)

Young adults fall into the trap of Big Tobacco’s promotional themes of independence and rebelliousness:

“when you are a child and you smoke…I am rebellious, I do what I want, look - I am smoking, I don’t care…I was a ‘bad girl’, I smoke, look at me” (F24-MIX)

but also into the belief that tobacco supports stress relief:

“it brings me some moment of quiet, for I take my cigarette and I think of my own problems and while it lasts, for 3 minutes it seems like nothing else matters…It’s just me and my cigarette…me aware of myself” (F24-MIX)

### People as influencers

The manner in which promotional teams, mainly represented by teams of two or more advertisers, almost all of them young girls, approached face-to-face encounters induced different reactions amongst young adults:

“all dressed alike and they don’t have the most intelligent faces and all of them, have the same words, for me it is amusing” (F19–CC) “I am impressed by the behaviour of….the ‘girls’, all dressed to the nines, they have elegant suits, behave professionally…they always hire pretty girls, always girls” (F24-MIX)

Phone calls provoke annoyance:

“most of the time [they] talk as if they’re robots….it’s very annoying” (F19–HTP)

Happy customers are excellent advocates and this applies to family and friends but also to huge celebrities or non-public figures.

“my father, immediately after I bought the IQOS came to me and asked “what is this?”, and after some days he bought his own device” (F21–HTP)

“using a movie star (…): she’s sexy and very beautiful, with a cigarette, inhaling the steam while you see the IQOS logo…” (M21-NS)

Some non-smoker young adults think that the vulnerable youth are being manipulated through advertising and marketing methods:

“I honestly saw smarter advertising methods in IQOS, more modern methods: influencers, Instagram, ads on social networks frequented by young people; using a movie star or a model…the youth are much easier to manipulate.” (M21-NS)

Some participants in our study felt the pressure and the aggressiveness of sales teams while others perceived this as sales perseverance:

“those promoters are very aggressive…they attack you in gas stations…it will come the moment when they will shove that device down our neck, probably” (F24–MIX)

“the girl needs to work and do her job, for this is the way she gets paid” (M20-MIX)

### Engagement with risk narratives

Our analysis found young adults had different types of engagement with risk narratives and this was likely to influence their decision to use HTPs. As no one in our sample started smoking using HTPs, but CCs, and as some of them are using both, we present data also on perceptions of combustion smoking risk.

### Smoking is about losing health

For the non-smokers, knowing the smoking risks and/or recognising the harmful effects in family members acted in some cases as reasons for not initiating or continuing smoking.

“… I think I was influenced because I saw my father when he had problems and he was choking or coughing. I was very young when these things happened, it was when he also tried to quit for the first time and I thought I didn’t want to be like him. I don’t know, probably being young the impact was bigger seeing my father so vulnerable”. (F21-NS)

For the smokers, we identified two types of answers: those affirming that smoking had harmful effects on health whilst those who doubted, dismissed, minimised or avoided engagement with risk narratives. One young adult, despite being a smoker, acknowledged smoking as a loss to health:

“Smoking, in general, cannot be healthy”. (M20-MIX)

Awareness of the harmful effects of smoking was based upon living with a long-term chronic respiratory condition, observations of the impact of smoking on their physical fitness and a desire to quit to avoid harm:

“I had asthma during my childhood so I should not smoke”. (F21-HTP)

“Before I start smoking my resistance to physical effort was better…after I started to smoke, I got tired quickly and feel like suffocating”. (M20-MIX)

“[I]considered quitting several times” (M20-MIX)

### Harmful effects of smoking - doubted, dismissed, minimised or avoided

However, for one participant, even when the harmful effects of smoking were acknowledged, this was qualified with the expression ‘at least’, used to reduce the effect of the statement of harm or indicate some doubt:

“I am aware it causes harm, at least this is what is said”. (F21-HTP)

Harmful effects of tobacco consumption were linked to the length of time someone had smoked. The expression *in time* was used to dismiss or minimise a statement of harm:

“Regarding smoking, people should not become dependent because in time addiction to tobacco cannot be healthy”. (M20-MIX)

“In time smoking can cause respiratory diseases, cancers etc”. (F21-HTP)

Similarly, reducing the amount of smoking activity was also seen as one way to minimise risks, with the belief ‘if I am not dependent, I am not at risk’:

“I am careful not to smoke too much…”. (M20-MIX)

Finally, young adults admitted to avoiding any engagement with risk narratives, by turning away from the pictures on packaging as risk reminders:

“In the beginning I saw them, now, after all these years they became normality and I do not feel affected so much, I mean I know that they are there but I cannot say that I see them anymore” (F25-MIX)

Diminishing, doubting, dismissing or even avoiding any engagement with risk narratives are in fact methods of self – rationalisation, well known strategies to diminish the discomfort of being psychologically incongruent when smoking while knowing in fact smoking is damaging health.

The way participants engaged with risk narratives was linked, in part, to the relationship within the family home (which we discuss further below, in relation to the social body, family bonds and a sense of an autonomous self).

### Perceptions of addiction

Very few young adults in our study shared knowledge of nicotine’s addictive mechanism:

“Anyway, it’s an addiction, it’s… Honestly, you get moody at some point if you don’t take your dose, so to speak” (F20-HTP)

Smoking was perceived more as a vice, rather than a behaviour related to a disease.

“Honestly, I don’t know…whether or not I will give up this vice” (F20-HTP)

Subsequently, smoking cessation was perceived as a matter of a *“very big will”* (M20-MIX) and not suitable for medical treatment and cessation support:

“Only the will, the iron will.” (F20-HTP)

Being considered a matter of will, none of the young adults mentioned the relevance of the combined cessation support in improving quitting effectiveness, which some seem to confuse with that offered by a friend:

“Yes, and my friend is trying to support me, “Leave it, leave it, leave it!”. (F20-HTP)

Some young adults considered they were not addicted:

“I do not smoke much, nor often”. (F21-HTP)

For others, the lack of withdrawal symptoms led them to believe they were not addicted:

“If I do not smoke [for a while] nothing happens to my body”. (M20-MIX)

### Risk narratives surrounding HTPs

When specifically asked about HTP smoking, most of the young adults acknowledged the same health risks whether they were tobacco smokers, HTPs users or non-smokers:

“They might contain fewer substances but they are equally causing harm”. (F21-HTP)

“Smoking, in general, cannot be healthy even if IQOS producers claim that it could be a healthier option”. (M20-MIX)

Others (smokers and non-smokers) considered that HTPs might present fewer health risks, or might be perceived as such:

“I understood they are less harmful”. (F20-HTP)

“People think they are somehow better than the others (traditional ones) and less harmful”. (F21-NS)

One participant declared that smoking HTPs seems different in terms of perceived harmful effects compared to regular cigarettes.

“No negative effects with IQOS until now. I do not cough at all. When I used to smoke traditional cigarettes, my teeth were yellow, with IQOS they look normal. IQOS leaves no specific taste and I do not think it affects taste as much as the traditional tobacco”. (M20-MIX)

Very few young adults in our study (2 of 9) described HTPs as being less harmful as their reason to switch from CCs to HTPs. One example is a participant who even perceived HTPs as enabling more physical activity:

“I suffered from Tetralogy of Fallot …This is the main reason I switched to HTPs…I couldn’t even run for 30 metres, but now I can even do sports” (F19-HTP)

### Social body, family bonds and autonomous self

One of the main themes of our research is how young adults’ risk behaviours and their engagement or resistance to marketing are influenced by their peer groups, family unit and/or individual sense of self. The latter self-concept fluctuated between a presentation of an autonomous self, unaffected by others, and a description of a social [not individual] body – in which the self is perceived as intricately bound and governed by cultural and social norms and the expectations of others.

### Individuality – autonomous self

Participants in our study saw HTPs as an opportunity to express and develop their felt sense of individuality, by seeking out novel experiences:

“The first time I tried IQOS…I received it when I celebrated my 18^th^ birthday; it had just appeared and everyone was wow!” (F20-HTP)

Novel experiences that challenged authority were seen as even more attractive:

“It was something ‘wow’ for me that I could hide from my parents”, therefore the thrill of being “afraid of getting caught” (F19-HTP)

Sensation seeking and curiosity are frequently expressed by many young adults as reasons to try smoking:

“I started smoking a little too early for what I should, more out of curiosity, somewhere around the age of 15” (F24-MIX)

“[Starting]…out of curiosity about the effect. Everyone felt more relaxed after smoking a cigarette. Everyone went out after school, before school, and they smoked. I say, what’s going on?” (F25-CC2)

Looking for new sensations and exploring what one’s self likes or dislikes can sometimes end either in fear of use or in rejection:

“[IQOS]…it seemed to be dangerous because it was heated… almost where the smoke was drawn, I always had the feeling that I’m going to get burned”. (F25-CC2)

“HTPs… I don’t even want to hear. The cigarette is a cigarette. I like the inhalation itself, but I still don’t like the smell. [HTP], that’s a steam device; how to like hot steam entering my throat?” (F26-CC)

Impulsive curiosity is frequently remembered:

“…it was just on the spur of the moment, an impulse of being young, but from that moment…it never stopped.” (F25-CC1)

Yet, a current non-smoker student in our study, educated and raised by non-smoking parents, described experimenting with smoking “just in order not to die stupid” (M18-NS). His behaviour was suggestive of a more cerebral, informed, or censored curiosity compared to the impulsive curiosity tied up with challenging authority figures. He, therefore, was able to override temptation and did not become a smoker.

For one participant, experiencing smoking is a facilitator for self-change - to become “*stronger but more determined, more assumed”* (F25-CC1):

“I thought that a certain category of kids was smoking and I would have liked to be like them. Personally, I am quite introverted and they were not like that;… they were more, I don’t know, popular, full of excitement” (F25-CC1)

For another young adult in our study, being authentic and living passionately, enjoying life experiences were important values she attributed to the experience of smoking. She described “*smoking* - a familiar companion of her childhood - *a way of living*”. (F19-CC)

### Family bonds

The family dynamic was clearly impacting upon young adults in our study and their smoking choices, including their engagement with marketing practices. Parents who smoked acted as role models: both positively and negatively. Young adults reported that if they had parents who smoked, despite their declared concern regarding the harmful health-related effects, their parents were ‘*pretty understanding’* regarding their child starting smoking:

“The idea that I had started smoking - was not good news for them but, in the end, they didn’t try very much to talk me out of it”. (F25-CC1)

“at 14 years old I didn’t tell them that I smoked, they found out eventually and it wasn’t all that good but it wasn’t all that bad either” (F19-CC)

Even more, parents purchased HTPs as birthday gifts for their adult children, illustrating the symbolic gift value attributed to a tobacco product for strengthening family bonds. For example,

“And I can remember that my mother gave it to me for my birthday, I remember it vividly” (F26-HTP)

Smoking together with parents, smoking the same tobacco brand at home or sharing cigars whilst having deep, reflective conversations, was mentioned by some of the young adults as significant events in their lives. For young adults, burdened by the uncertainty of their quests for the true self, such rituals facilitated a glimpse of real adulthood:

“doing what grown-ups do and feel” (F19-HTP).

These moments of child-parent bonding facilitated a perceived feeling of being seen and accepted as true individuals whilst also re-creating a feeling of safety and acceptance:

“I am good friends with my father and, in a very stupid way, when I sit at a cigarette with him I have the feeling that our friendship is strengthening …yes sharing and connecting” (F19-CC)

Daughters seem especially rewarded by smoking with their fathers - a sort of confirmation at the end of a rite of passage into adulthood:

“My dad told me that the first time we will smoke together will be on my 18th birthday” (F19-HTP).

For another female in our study, sharing the same CCs with her father was a bonding experience. This created a shared adult space, thus HTP marketing messages, designed to boost youthful social image, were of no interest to her, considering HTPs as a poor *“surrogate”*. (F19-CC)

Young adults who smoke, having parents who were smokers, demonstrate they remember their parents’ educational messages and mirror them by providing similar messages to their siblings:

“Don’t do it, it’s not OK [smoking], it’s not for you! I want the best for you and I’ll tell you what conclusion I came to - it’s not OK and it’s a waste of time, money, health” (F21-HTP)

Non-smoking parents were perceived by their non-smoking children not only as good role models but as authentic educators, succeeding in conveying pro-non-smoking messages that last:

“I was never interested in smoking. Honestly speaking, I think the reason has to do a lot with the education I received at home. With my parents being non-smokers, I was taught since I was little not to touch cigarettes as they are not pleasant… What also matters is how this educational message is delivered.” (M21-NS)

### Social body

Mirroring and adopting peer group behaviours was viewed by young adults in our study as a modality to anticipate acceptance, and the first step to bonding and belonging to a social group:

“Everybody was smoking, I needed to be there, along with my crowd. I have no idea what happened in my subconscious because I remember that I didn’t like to smoke, but… I kept going. It was something I needed to do because that was the reality, that was the thing that everybody did”. (F25-MIX)

Peer pressure is one of the most frequently cited reasons to give in to the temptation of smoking:

“At the beginning, I felt weird going out with them and not smoking; … They were asking me: why don’t you smoke? Give it a try! … It was mostly the pressure: see how it is, try it… plus a sort of curiosity to see how it is.” (F22-MIX)

At the same time, peer pressure was enough to override unpleasant first smoking experiences:

“First experience was terrible…the sensation, the taste, choking…and friends pressuring: try more, you are not inhaling deep inside…” (F19-HTP)

Therefore, peer pressure becomes a sort of instrument of giving up on the individual self to become part of the new (social body) entity. Similarly, pressure (via aggressive marketing) becomes an instrument of adherence towards the new culture- smoking in a group.

The power of the group and the importance of bonding with it was strengthened for young adults in our study by sharing CCs:

“I felt the need to offer my cigars to others; I like to share … because it’s not normal to smoke by yourself; the people around have to smoke as well” (F19-HTP)

Interestingly, in our data, HTP was not perceived as the ideal instrument for achieving the social body:

“… now I use HEETS and it’s not an option because not everyone has the device.” (F19-HTP)

## Discussions

The study focused on the influence that unregulated direct marketing of HTPs has on young Romanians regarding product knowledge, the initiation, and the use of HTPs. The data explored young adults’ experience with marketing strategies. A broad topic guide elicited insights into the decision to start smoking HTPs. Our study found three overarching themes: people, places and subjects of marketing, engagement with risk narratives and the role of the social body, family bonds and the autonomous self.

Most of the young adults, even the non-smokers, were fully aware of HTPs advertising, an aspect observed in other studies^30,31^. We organised young adults’ awareness of HTP advertising in terms of the seven P’s of tobacco marketing mix: Product, Price, Place, Promotion, People, Process and Physical evidence^26^. In our sample, a mix of marketing elements was acknowledged: the product’s image and price, promotion strategies used by sales teams and advertising places. Young adults’ perception is that tobacco marketing is selling a range of HTP devices: technically appealing, associating positive qualities (elegant-engaging, picturing a certain lifestyle) and potentially less risky for health (although young adults are ambivalent related to this claim). They are ideal for indoor usage and, on the long run, have lower costs than CCs. Free samples and gratuity packages (“two for the price of one”) were seen as very attractive, the financial hook being reported in other works^15^. In the short term, HTPs are perceived as expensive, hence one potential reason not to start smoking by using HTP. Another reason for not starting smoking HTPs could be the perceived barrier in sharing an HTP device, therefore a less ideal bonding instrument compared to CCs. Everyone in our study had been approached by promoters or acknowledged social media influencers, these being known methods of marketing^20,32^. From shopping, recreational venues, and events, to the unlimited space of the internet^33,34^, day-to-day life seems impregnated by tobacco products.

Yet, participants in our study were not aware of the marketing influence, declaring that starting or continuing smoking was only about their own will, their own decision. This contrasts existing data demonstrating tobacco’s influence on young adults’ smoking^35,36^. This might be explained by self-rationalisation^37–39^. In order not to feel guilty or disempowered, young adults develop strategies to downplay the influence of marketing by either diminishing, minimising, or casting doubt on the harm produced by smoking CC or HTP use. To combat this self-rationalisation, more policy and public health driven measures for a de-marketing tobacco strategy are needed. Such a de-marketing strategy would take each component of the 7 Ps of the marketing mix described in our results and create a de-marketing strategy^40^, replicating work done in other regions.

All the young adults we interviewed were aware of the smoking risks. These findings are in line with the results of a previous study, published in 2018, where Romania had one of the highest indexes of knowledge of health risks of smoking^30^. However, we observed that contrary to the results in other studies^12,31,33^, when specifically asked about HTP use, most of the respondents (HTP users or non-smokers) were ambivalent, while some didn’t acknowledge a reduced health risk of HTPs compared to CCs. We also identified the risk was perceived differently on general and personal levels, as the youth considered themselves not being at risk (they equated the harmful effects of tobacco consumption with frequency and amount of consumption), whilst advocating scepticism with suppliers claims that HTPs were less harmful.

The triadic influence theory^36,41^, a framework developed to explain substance use in adolescents, states that health-related behaviours are influenced by three “ultimate causes” (a person’s general cultural environment, the current social situation, and personal characteristics) via three streams of factors ending with proximal determinants, respectively the attitudes, the social normative pressures to perform health-related behaviours and the perceptions of self-efficacy^41^.

In this article, we have provided data on all three, but particularly concerning the general cultural environment and the social context.

General/cultural environment. Integrating HTP marketing in the general landscape and making it ubiquitous (Places and People - Influencers) is a method of normalising HTP use and shaping smoking-related attitudes: HTPs could be more acceptable since the product is presented as having more favourable consequences on personal (less harmful), financial (less expensive on the long run) and social level (appealing, good group integration facilitator).

Social Context. Social influences play a major role in health-related behaviours in general, and substance use in particular^41^.

Individual level. On the individual level, direct marketing methods elicit strong emotions that can be followed depending on the perceived self-efficacy^42^ by a decision and a behavioural response: to “buy and use” or to “don’t give up – i.e. do not use/smoke”. According to Bandura, since “health perceived self-efficacy depends on the will to control behaviours in general and the perceptions of personal skills in controlling the behaviour, people who have the will to control their behaviours and believe they have the skills to perform a certain behaviour should have stronger health-related self-efficacy and should be more likely to decide to adopt health-promoting behaviours”^42^.

According to the Triadic Influence Theory^41^, decisions taken by young people are subjectively rational, not “objectively rational”, they do not have the capacity and rigour to take into consideration all possible consequences of the behaviour or the alternative benefits of the alternative behaviour, due to neurodevelopmental specificities of the age, personality characteristics as well as social normative beliefs and cultural shaped attitudes towards the respective behaviour^42^.

Whether it is a striking graphic design (elegant and colourful) or a surprising new electronic device, with a technological appeal, there are emotions that drive purchasing behaviour/decision^43^, with all the components: alternatives, interpersonal issues, high-risk consequences, and uncertainty. Tobacco producers, understanding young adults’ biological vulnerability, target and imprint them with all the elements of the marketing mix, namely the product design, packaging, promotions, pricing, and distribution networks^26^. Young people are well aware of all these aspects.

On the other hand, demarketing strategies also use emotions (i.e. depicting images of smoking-related diseases on the packaging) in an attempt to increase smoking awareness and cessation^44,45^.

In our data, we identified a fluctuation between the presence of an autonomous self, unaffected by others and the description of a social body in which the self is perceived as intricately bound and governed by cultural and social norms and the expectations of others. Young adults in our study saw HTPs as an opportunity to express and develop their felt sense of individuality, by seeking out novel experiences, and being led by a whole range of expressions of curiosity (i.e. impulsive to more cerebral/controlled curiosity). According to Siegel^33^, changes in the fundamental circuits of the brain during adolescence explain the increased drive for reward, resulting in increased impulsiveness and susceptibility to addiction. Yet, none of our participants seemed to know nicotine’s addictive mechanism and therapeutic support options for smoking cessation, considering smoking more a vice than a disease and smoking cessation a matter of will and not subject to medical treatment and support. Literature^42,46^ also depicts adolescents’ proclivity to ‘hyperrationality’ - downplaying the significance of a negative outcome while amplifying the significance given to a positive result, which explains teen’s tendency to risky behaviours and the preference for immediate rewarding present, against the possible future harms.

In a period when breaking dependence from family (supported by biological development) becomes vital for self-quest initiation, the peer-group integration challenges individuals to respect ‘the game’ (doing what others do, to smoke). The very act of smoking is perceived by young adults as either ‘too daring’, therefore somehow shameful in report with family rules, or a symbol of conformity, reported to peer-group rules. Interestingly, according to the literature, adolescents’ proclivity to risk-taking is amplified by being in a group of peers^46^.

Being accepted by family, peers and community is of paramount importance for young adults in such a vulnerable developmental period^47^. In our data, young adults manifested a strong interest in smoking as an important facilitating instrument for acceptance by the peer group. According to young adults, parents who smoke perceived smoking HTPs or CCs as either being ‘quite acceptable’, or invested with ritualic power (i.e. creating a ‘shared adult space’ or a rite of passage celebration). The family of smokers implicitly ‘normalise’ the habit, while tobacco marketing normalises smoking on purpose. Such ‘normalising’ cultural and social environments seem to challenge young adults’ biological vulnerability and result in trial and eventually reinforced HTP use or CC smoking behaviour.

Our qualitative methodology allowed us to explore perceptions, opinions, and personal experiences of the participants. We had three researchers collecting the data and we consider this as a strength because it facilitated a lively interaction in data sharing meetings. To limit the variability that might appear when more interviewers participate in the same project, we relied on the developed topic guide and discussions before the start of the interviews. At the same time, having multiple interviewers allowed us to reach saturation with a lower sample of participants than initially expected. Another strength is that the data was double coded.

We recognise that our analysis is mainly limited to HTPs users who continue to use also CCs. We did not manage to recruit young adults starting smoking as HTP users, which, on its own, was an interesting finding; a different recruitment strategy could have identified HTP-only users.

Some other limitations were due to the pandemic context (lockdown): conducting the interviews online may have limited interactions. The pandemic and social isolation policies will have impacted young adults’ recent direct contact with marketing places and people, impacting on what they recalled during interviews.

We observed a higher proportion of females that participated in the study, even though according to existing epidemiological data from Romania, men have a significantly higher prevalence of smoking^48^. This might prove as a limitation for the transferability of the results, as gender could play a role in different reasons for using HTP^49^. At the same time, participants in the study did not acknowledge the presence of gender-oriented marketing strategies for HTP. Most of the sample were trainee doctors (12 participants out of 19). This may limit the transferability of our findings to the wider young adult population in Romania, especially in relation to the results referring to the engagement with the risk narratives.

We observed that trainee doctors are actively diminishing, minimalising or doubting the harm produced by tobacco (either smoking of CC or HTP use). This raises two important concerns for future research. First, how does the general population of young adults diminish, minimalise or doubt risk narratives compared to medical trainees and second, what impact, if any, do the diminishment, minimalisation and doubt of tobacco and HTP risk narratives have on future smoking cessation interventions for their patients?

Taking advantage of the gap in the specific legislation, which bans the advertising of products that burn, but not of those that heat tobacco, HTPs are intensely marketed among young adults as a less harmful alternative to smoking, using promotion strategies aiming to infiltrate the social activities and normalise tobacco use. Despite recognising pieces of the industry’s complex strategy, young adults do not acknowledge the big picture and (HTP/CC) the marketing influence on their decision to experience smoking. They manifest ambivalence regarding HTPs’ potentially less harmful effects while emphasising the instrumental role of tobacco usage in bonding with peers and parents who smoke. Hence, a direct practical implication of our study is how educationalists and clinicians should approach conversations with young adults (and their parents) about lung health and HTP, particularly in the Romanian context. Therefore, all the preventive interventions (Very Brief Advice included^50^) should refer also to the risks associated with HTP use and support this with information sheets describing the present lack of scientific evidence regarding the known harms of HTPs.

## Supplementary information

Reporting Summary

## Data Availability

The data that support the findings of this study are available from the corresponding author (SC) upon request.
