# Supplementary material for: Romanian young adult perceptions on using heated tobacco products following exposure to direct marketing methods
Source: NPJ Prim Care Respir Med. 2023 Mar 2;33:8. doi: 10.1038/s41533-023-00333-y (PMC9981255; doi:10.1038/s41533-023-00333-y)
Supplement: Supplementary file 1 — Reporting Summary [file 41533_2023_333_MOESM1_ESM.pdf]

nature portfolio

Corresponding author(s): Sergiu Chirila
Last updated by author(s): Feb 15, 2023

Reporting Summary

Nature Portfolio wishes to improve the reproducibility of the work that we publish. This form provides structure for consistency and transparency in reporting. For further information on Nature Portfolio policies, see our Editorial Policies and the Editorial Policy Checklist.

Statistics

For all statistical analyses, confirm that the following items are present in the figure legend, table legend, main text, or Methods section.

n/a	Confirmed	
☐	☒	The exact sample size (*n*) for each experimental group/condition, given as a discrete number and unit of measurement
☒	☐	A statement on whether measurements were taken from distinct samples or whether the same sample was measured repeatedly
☒	☐	The statistical test(s) used AND whether they are one- or two-sided<br>*Only common tests should be described solely by name; describe more complex techniques in the Methods section.*
☒	☐	A description of all covariates tested
☒	☐	A description of any assumptions or corrections, such as tests of normality and adjustment for multiple comparisons
☒	☐	A full description of the statistical parameters including central tendency (e.g. means) or other basic estimates (e.g. regression coefficient) AND variation (e.g. standard deviation) or associated estimates of uncertainty (e.g. confidence intervals)
☒	☐	For null hypothesis testing, the test statistic (e.g. *F*, *t*, *r*) with confidence intervals, effect sizes, degrees of freedom and *P* value noted<br>*Give P values as exact values whenever suitable.*
☒	☐	For Bayesian analysis, information on the choice of priors and Markov chain Monte Carlo settings
☒	☐	For hierarchical and complex designs, identification of the appropriate level for tests and full reporting of outcomes
☒	☐	Estimates of effect sizes (e.g. Cohen's *d*, Pearson's *r*), indicating how they were calculated

Our web collection on statistics for biologists contains articles on many of the points above.

Software and code

Policy information about availability of computer code

Data collection	No software was used
Data analysis	No software was used

For manuscripts utilizing custom algorithms or software that are central to the research but not yet described in published literature, software must be made available to editors and reviewers. We strongly encourage code deposition in a community repository (e.g. GitHub). See the Nature Portfolio guidelines for submitting code & software for further information.

Data

Policy information about availability of data

All manuscripts must include a data availability statement. This statement should provide the following information, where applicable:

- Accession codes, unique identifiers, or web links for publicly available datasets
- A description of any restrictions on data availability
- For clinical datasets or third party data, please ensure that the statement adheres to our policy

The data that support the findings of this study are available from the corresponding author (SC) upon request.

Human research participants

Policy information about studies involving human research participants and Sex and Gender in Research.

Reporting on sex and gender	Sex was determined by self-reporting. On analyzing the demographics of the participants, sex was used. When analyzing the generated data, we used gender, as it offers the integration with the social and cultural circumstances.
Population characteristics	See above.
Recruitment	Participants filled in an on-line questionnaire where, the ones willing to participate in the interview provided their contact data. To complete the study sample, some participants were invited to the interviews based on snowball sampling.
Ethics oversight	Ovidius University of Constanta

Note that full information on the approval of the study protocol must also be provided in the manuscript.

Field-specific reporting

Please select the one below that is the best fit for your research. If you are not sure, read the appropriate sections before making your selection.

☐ Life sciences ☒ Behavioural & social sciences ☐ Ecological, evolutionary & environmental sciences

For a reference copy of the document with all sections, see nature.com/documents/nr-reporting-summary-flat.pdf

Behavioural & social sciences study design

All studies must disclose on these points even when the disclosure is negative.

Study description	The data reported in this study is qualitative.
Research sample	Romanian young adults, aged 18-26, stratified based on their tobacco use status and type, in four groups> combustible cigarettes smokers, heated tobacco product users, mixt users and non-smokers. This stratification aimed at obtaining an as large and detailed palette of opinions as possible.
Sampling strategy	We aimed to recruit between 25 and 30 young adults to be interviewed, using a purposive sampling strategy, based on the initial survey. We combined the purposive sample strategy with snowball sampling.<br>We stopped at 19 interviews, from the planned 25-30, because we felt that data saturation with respect to our aim was achieved.
Data collection	The interviews were conducted online, using Zoom teleconference platform. They followed a topic guide developed by the researcher and were recorded audio and video. In the interview only the researcher and the participant were present.
Timing	All interviews were conducted online between May 2020 to February 2021
Data exclusions	No data were excluded from the analysis.
Non-participation	No participant dropped out from the study.
Randomization	There were no experimental groups. Participants were grouped according to their smoking status in order to better illustrate young adults experiences.

Reporting for specific materials, systems and methods

We require information from authors about some types of materials, experimental systems and methods used in many studies. Here, indicate whether each material, system or method listed is relevant to your study. If you are not sure if a list item applies to your research, read the appropriate section before selecting a response.

Materials & experimental systems

n/a	Involved in the study
☒	☐ Antibodies
☒	☐ Eukaryotic cell lines
☒	☐ Palaeontology and archaeology
☒	☐ Animals and other organisms
☒	☐ Clinical data
☒	☐ Dual use research of concern

Methods

n/a	Involved in the study
☒	☐ ChIP-seq
☒	☐ Flow cytometry
☒	☐ MRI-based neuroimaging
